# Supplementary material for: Spike processing with a graphene excitable laser
Source: Sci Rep. 2016 Jan 12;6:19126. doi: 10.1038/srep19126 (PMC4709573; doi:10.1038/srep19126)
Supplement: Supplementary Information [file srep19126-s1.pdf]

Supplementary Information: Spike processing with a graphene excitable laser

B. J. Shastri,* M. A. Nahmias,* A. N. Tait,* A. W. Rodriguez, B. Wu, and P. R. Prucnal†
Department of Electrical Engineering, Princeton University, Princeton, New Jersey 08544, USA

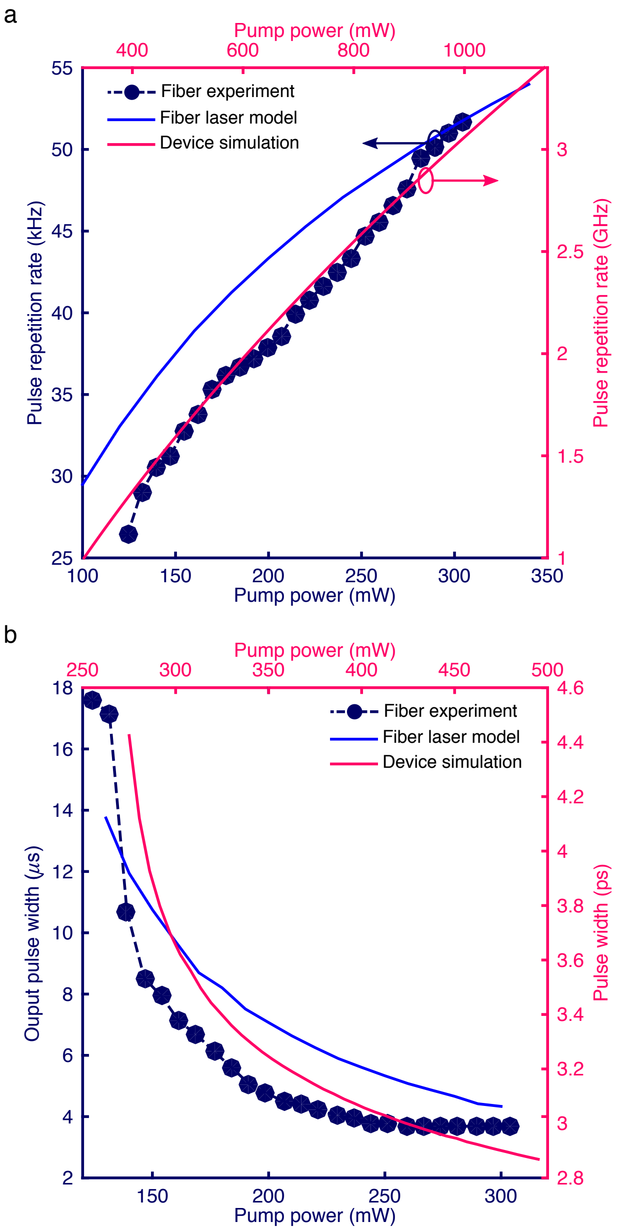

Supplementary Figure S 1. Typical characteristics of the passively Q-switched fiber and integrated lasers. (a) Output pulse repetition rate and (b) pulse width as a function of the pump current.

* These authors contributed equally to this work.

† Correspondence and requests for materials should be addressed to B.J.S. (shastri@ieee.org) or P.R.P. (prucnal@princeton.edu)
